# Insights into a dinoflagellate genome through expressed sequence tag analysis

**DOI:** 10.1186/1471-2164-6-80

**Published:** 2005-05-29

**Authors:** Jeremiah D Hackett, Todd E Scheetz, Hwan Su Yoon, Marcelo B Soares, Maria F Bonaldo, Thomas L Casavant, Debashish Bhattacharya

**Affiliations:** 1 Department of Biological Sciences and Roy J. Carver Center for Comparative Genomics, University of Iowa, Iowa City, IA 52242, USA; 2 Department of Ophthalmology and Center for Bioinformatics and Computational Biology, University of Iowa, Iowa City, IA 52242, USA; 3 Department of Pediatrics, University of Iowa, Iowa City, IA 52242, USA; 4 Departments of Biochemistry, Orthopaedics, Physiology, and Biophysics, University of Iowa, Iowa City, IA 52242, USA; 5 Department of Electrical and Computer Engineering, University of Iowa, Iowa City, IA 52242, USA

## Abstract

**Background:**

Dinoflagellates are important marine primary producers and grazers and cause toxic "red tides". These taxa are characterized by many unique features such as immense genomes, the absence of nucleosomes, and photosynthetic organelles (plastids) that have been gained and lost multiple times. We generated EST sequences from non-normalized and normalized cDNA libraries from a culture of the toxic species *Alexandrium tamarense *to elucidate dinoflagellate evolution. Previous analyses of these data have clarified plastid origin and here we study the gene content, annotate the ESTs, and analyze the genes that are putatively involved in DNA packaging.

**Results:**

Approximately 20% of the 6,723 unique (11,171 total 3'-reads) ESTs data could be annotated using Blast searches against GenBank. Several putative dinoflagellate-specific mRNAs were identified, including one novel plastid protein. Dinoflagellate genes, similar to other eukaryotes, have a high GC-content that is reflected in the amino acid codon usage. Highly represented transcripts include histone-like (HLP) and luciferin binding proteins and several genes occur in families that encode nearly identical proteins. We also identified rare transcripts encoding a predicted protein highly similar to histone H2A.X. We speculate this histone may be retained for its role in DNA double-strand break repair.

**Conclusion:**

This is the most extensive collection to date of ESTs from a toxic dinoflagellate. These data will be instrumental to future research to understand the unique and complex cell biology of these organisms and for potentially identifying the genes involved in toxin production.

## Background

Dinoflagellates play critical roles in marine ecosystems as primary producers and grazers of other bacterial and eukaryotic plankton [1]. Approximately one-half of the ca. 4,000 species of dinoflagellates contain plastids, although many are mixotrophic [2]. Many taxa produce potent toxins and form harmful algal blooms, or "red tides", resulting from populations of more than 20 million cells per liter of seawater. The toxins cause a variety of poisonings that affect humans and marine wildlife [1] and have a significant impact on coastal ecosystems throughout the world [3]. Yet, other dinoflagellates, like *Symbodinium*, are central contributors to the health of reef ecosystems as the symbionts of corals [4]. Loss of the dinoflagellate symbiont results in coral bleaching. In addition to their ecological role, dinoflagellates display some fascinating and unique aspects of cell biology. One intriguing character is nuclear biology. The nucleus of dinoflagellates is unlike that of any other eukaryote because the chromosomes are condensed throughout the cell cycle except during DNA replication [5]. The morphologically similar chromosomes are attached to the nuclear envelope and can number in the hundreds [6]. Dinoflagellates also lack nucleosomes [7], instead the nuclear DNA is associated with basic proteins that are moderately similar to bacterial histone-like proteins (HLPs [8,9]). Dinoflagellates were thought to lack histones [10], but in a recent gene expression study, a putative histone H3 was annotated in *Pyrocystis lunula*, although the sequence was not analyzed further [11]. The general lack of nucleosomes raises many questions about transcription and gene regulation in these organisms. Dinoflagellate nuclei also contain vast amounts of DNA compared to other eukaryotes. Estimates range from 3 – 250 pg·cell^-1^, or approximately 3,000 – 215,000 megabases (MB) [12]. In comparison, human nuclei contain 3.2 pg·cell^-1 ^(3,180 MB). The dinoflagellate nucleus contains such a high concentration of DNA that it exists in a liquid crystal state, which is responsible for the unique morphology [13,14]. The DNA to basic protein ratio of dinoflagellate chromosomes has been estimated to be 10:1, which is dramatically higher than the 1:1 ratio observed in most eukaryotes. This indicates that very little basic protein is associated with dinoflagellate chromosomes and that the crystal structure is the primary cause of the unusual morphology. Dinoflagellates are also the only eukaryotes to contain hydroxymethyluracil, a deaminated nucleotide that can be produced by oxidative damage of DNA, which replaces 12 – 70% of the thymidine [15]. The role of polyploidy or potentially, genome amplification within particular life history stages remains to be clarified for dinoflagellates. It is highly unlikely, however, given their relatively simple morphology that the immense DNA content is explained solely by gene content.

The most widespread plastid in dinoflagellates contains the unique photopigment peridinin. The "peridinin plastid" is remarkably different from this organelle in other eukaryotes because it lacks a typical genome. Plastids normally contain a circular genome of about 150 kb that encodes 100 – 200 genes that are necessary for plastid function. In peridinin-containing dinoflagellates, the plastid genome has been broken into minicircles that encode a single, or a few genes per circle. However, only 16 genes have been identified thus far on minicircles [16,17]. Recent studies show that most of the plastid genes have been transferred to the nucleus [18,19] with 15 of these genes found exclusively on the plastid genome in all other photosynthetic eukaryotes [18]. The peridinin dinoflagellates encode therefore the smallest number of plastid genes of any photosynthetic eukaryote, making them a model for understanding organellar gene transfer. Nuclear-encoded plastid proteins are targeted to the plastid using a tripartite N-terminal targeting signal [20]. As in *Euglena*, nuclear-encoded plastid proteins are co-translationally inserted into the endoplasmic reticulum and embedded in this membrane using a stop-transfer sequence in the N-terminus. Through algal endosymbioses, the dinoflagellates have been able to acquire four other types of plastids from distantly related evolutionary lineages including the haptophytes, cryptophytes, diatoms, and prasinophytes [1,21]. This aspect of their evolutionary history highlights the unmatched ability of dinoflagellates to capture and retain foreign plastids.

*Alexandrium tamarense *is one of the best-studied dinoflagellates. This species forms toxic blooms and causes paralytic shellfish poisoning through saxitoxin production. It has a peridinin-containing plastid and in North America, *A. tamarense *blooms from Alaska to Southern California in the Pacific and along the Canadian and New England coasts in the Atlantic. There has been a recent increase in blooms of *A. tamarense *and other *Alexandrium *species in other parts of the world making this genus of high importance to the world's fisheries. We undertook a gene discovery project with this organism using expressed sequence tag (EST) data to investigate dinoflagellate evolution and to create a genomic resource for scientists working on different aspects of *A. tamarense *and dinoflagellate biology. The EST method was the most reasonable approach in this case because haploid *A. tamarense *cells contain approximately 143 chromosomes and have a genome size of 200 pg/cell (ca. 200,000 Mb [Erdner and Anderson unpublished data]). Our EST results comprise the first extensive high-throughput, genome-wide data set for a dinoflagellate.

## Results and discussion

### Clustering and sequence analyses

**Figure 1:**
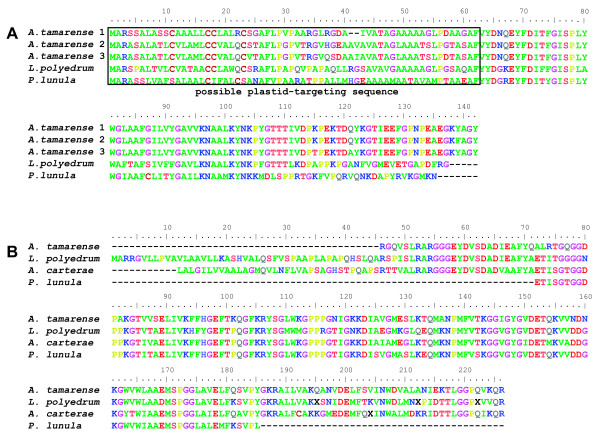
**Putative dinoflagellate-specific proteins. **Amino acid sequence alignments of putative dinoflagellate-specific proteins. A) putative plastid protein that was highly represented in the *A. tamarense *cDNA library (cluster size = 22). *A. tamarense *sequences 1, 2, and 3 correspond to clones GC1-aba-e-13, GC1-abh-e14, and GC1-abd-o-22, respectively, and are aligned with highly similar ESTs from the dinoflagellates *L. polyedrum *(CD809498) and *P. lunula *(BU582532). The boxed region indicated a possible plastid targeting sequence. B) Putative dinoflagellate specific protein with significant blast hits only to other dinoflagellate ESTs. The *Alexandrium *sequence corresponds to clone UI-D-GC1-abh-f-23-0-UI.

The collection of 11,171 ESTs comprised of single-pass 3'-reads (483 from the start library and 10,688 from the normalized library) from *A. tamarense *was assembled into 6,723 clusters. The normalized library showed a high degree of complexity, with a novelty rate of 60.18% and about 52% of the sequenced clones contained inserts that were longer than the single sequence read (ca. 750 bp). Clustering of the total EST set showed that most of the reads were singletons (4,618 sequences) and the largest cluster was comprised of 46 ESTs that are closely related to HLPs (Table 1). Other highly represented transcripts were those encoding luciferin-binding protein (a protein involved in the regulation of bioluminescence) and several photosynthetic proteins (e.g., Rubisco, ATP synthase C chain, light harvesting proteins). Several large clusters were transcripts that lacked a similarity (e-value < e^-5^) to known proteins. One of these ESTs has an open reading frame that encodes a protein with a potential plastid-targeting signal (Figure 1A). Interestingly, database searches against NCBI's nr and dbEST returned hits only to other dinoflagellate ESTs. Another of the largest clusters only had hits to ESTs from other dinoflagellates (Figure 1B). These two proteins are therefore candidates for dinoflagellate-specific proteins.

**Table 1:** Cluster size and frequency of the *A. tamarense *ESTs.

Cluster Size	Frequency	Cluster Size	Frequency	Best BLAST hit(s)
1	4618	14	7	
2	1249	15	1	unknown
3	427	16	1	HSP90
4	176	17	4	peridinin-chl a protein, Cytochrome C6, EF1-alpha, unknown
5	81	18	1	ATP synthase C chain
6	44	19	2	Form II Rubisco, unknown putative dino. specific protein
7	32	21	1	fucoxanthin chlorophyll a/c binding protein like
8	15	22	1	Unknown putative plastid protein
9	21	23	1	Unknown
10	13	24	3	peridinin-chlorophyll a protein, ATP synthase C chain, unknown
11	10	29	1	luciferin-binding protein
12	7	46	1	histone-like protein/basic nuclear protein
13	6			

**Figure 2:**
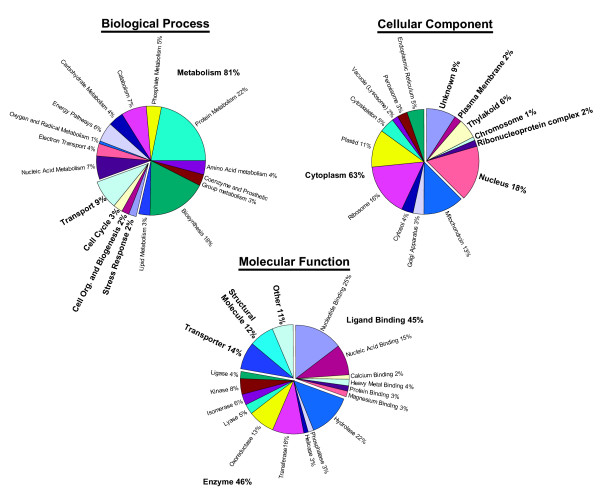
**GO category assignment of *A. tamarense *ESTs. **Classification of 1,203 *A. tamarense *ESTs into the GO categories.

Each cluster was searched against the SwissProt protein database using blastx. A total of 515 hits with an e-value less than 1e-20 were identified that terminated within 10 amino acids of the end of the SwissProt entry. From these hits, we estimated that the 3'-UTRs ranged in length from 25 – 620 nt with a mean length of 155 nt. This is shorter than the average length observed for fungi (~200 nt) and metazoans (300–600 nt) [22]. However, this analysis is likely to be an underestimate of the average 3'-UTR length because only ESTs that were sequenced into the coding region were included in the analysis. The 3'-UTRs of *A. tamarense *cDNAs are also interesting because of their apparent lack of a polyA signal. Both simple n-mer searches (e.g. hexamer, pentamer) and the Gibb's sampler were used to assay the canonical region from -11 to -30 preceding the polyadenylation site in search of a polyadenylation signal. We were unable to find a single or a related set of hexamers or pentamers that are enriched in the 3'-UTRs (data not shown). Clearly, polyadenylation of transcripts occurs in *A. tamarense*, however, the mechanism by which this process takes place apparently does not involve a typical polyA signal. These ESTs were also analyzed for GC-content and codon usage. Coding region GC-content was 60.8%, whereas GC-content in the 3'-UTR was slightly less at 57.6%. The GC-content is reflected in the codon usage (Table 2), whereby 3^rd ^positions are strongly biased towards Gs or Cs. The stop codon TGA is also significantly favoured over TAG and TAA (frequencies of 411, 71, and 25 occurrences, respectively). The accession numbers of SwissProt hits with an e-value of 9e^-10 ^and below (1,292 sequences) were submitted to the ProToGo server for GO category assignment [23]. A total of 1,203 of the SwissProt accession numbers could be assigned to GO categories. The results are summarized in Figure 2. The functional distribution of the *A. tamarense *ESTs that could be placed among GO categories is typical of other eukaryotes. However, the overall small number (i.e., 20%) of significant hits to GenBank is surprising, suggesting that many *A. tamarense *proteins may be either highly diverged and/or encode novel dinoflagellate-specific functions (e.g., Figure 1), or the sequence does not extend into the coding region of the transcript.

**Table 2:** Codon Usage in the *A. tamarense *ESTs.

TTT F	703	23.1%	TCT S	482	10.0%	TAT Y	372	18.8%	TGT C	251	15.6%
TTC F	2335	76.9%	TCC S	1348	27.9%	TAC Y	1612	81.2%	TGC C	1356	84.4%
TTA L	61	0.9%	TCA S	413	8.6%	TAA *	29	5.6%	TGA *	411	79.8%
TTG L	1118	15.7%	TCG S	926	19.2%	TAG *	75	14.6%	TGG W	1051	100.0%
											
											
CTT L	902	12.7%	CCT P	751	17.6%	CAT H	464	25.7%	CGT R	475	9.6%
CTC L	2296	32.3%	CCC P	1382	32.4%	CAC H	1340	74.1%	CGC R	1779	35.8%
CTA L	139	2.0%	CCA P	829	19.4%	CAA Q	433	14.6%	CGA R	426	8.6%
CTG L	2596	36.5%	CCG P	1307	30.6%	CAG Q	2535	85.4%	CGG R	1128	22.7%
											
											
ATT I	715	19.1%	ACT T	542	13.1%	AAT N	508	21.0%	AGT S	344	7.1%
ATC I	2770	74.1%	ACC T	1442	34.9%	AAC N	1915	79.0%	AGC S	1310	27.2%
ATA I	253	6.8%	ACA T	638	15.4%	AAA K	415	8.5%	AGA R	253	5.1%
ATG M	2096	100.0%	ACG T	1510	36.5%	AAG K	4485	91.5%	AGG R	910	18.3%
											
											
GTT V	686	11.2%	GCT A	1195	15.2%	GAT D	1117	24.9%	GGT G	943	13.8%
GTC V	2214	37.8%	GCC A	2899	36.8%	GAC D	3371	75.1%	GGC G	3957	58.1%
GTA V	268	4.6%	GCA A	1559	19.8%	GAA E	750	13.8%	GGA G	767	11.3%
GTG V	2694	46.0%	GCG A	2218	28.2%	GAG E	4682	86.2%	GGG G	1142	16.8%

Analysis is of 515 proteins (81,893 codons). Third position nucleotide usage was T = 12.8%, A = 9.3%, C = 40.7%, G = 37.2%. The asterisk (*) indicates a stop codon.

**Figure 3:**
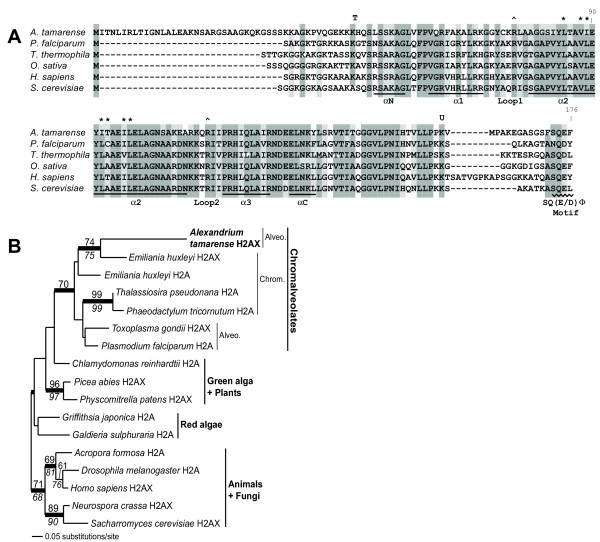
**Analyses of *A. tamarense *histone H2A.X. **A) Alignment of *A. tamarense *H2A.X with eukaryotic homologs. The alignment is shaded according to the level of conservation. The symbols above the alignment indicate the location of functional residues (T = trypsin cleavage site, ^ = arginines that contact the DNA helix, * = H2A-H2B interaction sites, U = ubiquitination site). The annotation below the alignment indicates conserved structural features including the α-helices, loops, and the SQ(E/D)Φgotif. B) A ML tree of H2A and H2A.X. The numbers above and below the branches are the results of ML and NJ bootstrap analyses, respectively. The thick branches indicate > 0.95 posterior probability from Bayesian inference. Only bootstrap values ≥ 50% are shown. Branch lengths are proportional to the number of substitutions per site (see scale bar).

### Dinoflagellate gene content and gene families

Of species with sequenced genomes, the apicomplexan *Plasmodium falciparum *is the most closely related organism to *A. tamarense*. Both of these species are members of the alveolate lineage with dinoflagellates and apicomplexans forming a monophyletic clade that is sister to the ciliates (e.g., [23]). Sequence comparisons using BLAST revealed that 609 of the 6723 *A. tamarense *ESTs had a significant hit (e-value less than 1e^-10^) to *P. falciparum *proteins. The top 20 most significant hits are shown in Table 3. The most highly conserved proteins between these organisms include many "housekeeping" proteins such as α-tubulin and heat shock protein 70. Despite their close evolutionary relationship, there are however likely to be substantial differences between *A. tamarense *and *P. falciparum *with respect to gene content. Due to the apicomplexan intracellular lifestyle, *P. falciparum *has lost most of the genes related to plastid function as well as other metabolic genes. Many of these same proteins appear in the list of the top BLAST hits against the nr database of GenBank (Table 4). There were 1,349 hits to the nr database that were better than 1e^-10^.

**Table 3:** Top 20 *A. tamarense *EST blast hits against the genome of the apicomplexan *P. falciparum*.

***A. tamarense *EST**	**E-Value**	**GI Number**	**Protein Description**
UI-D-GC1-aao-m-13-0-UI	1.00E-112	23613558	α-tubulin
UI-D-GC1-aav-f-09-0-UI	6.00E-86	23508137	flavoprotein subunit of succinate dehydrogenase
UI-D-GC1-aad-d-15-0-UI	9.00E-86	23509363	serine/threonine protein phosphatase
UI-D-GC0-aae-b-08-0-UI	2.00E-85	23509135	actin
UI-D-GC1-aaz-h-12-0-UI	3.00E-85	23507885	26S proteasome regulatory subunit 4
UI-D-GC1-abe-o-23-0-UI	8.00E-85	23510155	bifunctional dihydrofolate reductase-thymidylate synthase
UI-D-GC1-abh-e-16-0-UI	1.00E-84	23612827	hsp70
UI-D-GC0-aae-p-02-0-UI	2.00E-84	23613232	adenosylhomocysteinase
UI-D-GC1-aay-i-10-0-UI	3.00E-82	16804988	helicase
UI-D-GC1-aau-b-16-0-UI	1.00E-80	23509325	eukaryotic translation initiation factor 2 gamma subunit
UI-D-GC0-aae-h-03-0-UI	8.00E-78	23509820	glyceraldehyde-3-phosphate dehydrogenase
UI-D-GC1-aao-o-20-0-UI	4.00E-77	23508006	ADP ribosylation factor 1
UI-D-GC0-aae-f-01-0-UI	1.00E-76	23509545	calmodulin
UI-D-GC1-abb-n-18-0-UI	2.00E-76	23510206	eukaryotic initiation factor
UI-D-GC1-abf-g-07-0-UI	4.00E-75	23612467	HSP86
UI-D-GC1-abd-m-07-0-UI	2.00E-74	23612587	40S ribosomal protein S5
UI-D-GC0-aae-b-08-0-UI	3.00E-74	23509345	actin II
UI-D-GC1-aab-m-24-0-UI	4.00E-74	23509670	ribosomal protein S2
UI-D-GC1-aar-f-11-0-UI	3.00E-72	23509852	protein serine/threonine phosphatase
UI-D-GC1-aao-b-16-0-UI	1.00E-69	23509877	RNA helicase 1

**Table 4:** Top 20 hits of the *A. tamarense *ESTs to the GenBank nr database.

***A. tamarense *EST**	**E-Value**	**GI Number**	**Protein Description**	**Organism**
UI-D-GC1-abg-i-22-0-UI	1.00E-110	845405	ribulose 1,5-bisphosphate carboxylase	*Gonyaulax polyedra*
UI-D-GC1-aao-m-13-0-UI	1.00E-109	135433	alpha tubulin	*Oxytricha granulifera*
UI-D-GC1-abh-e-16-0-UI	2.00E-98	20143982	hsp70	*Crypthecodinium cohnii*
UI-D-GC1-abe-o-23-0-UI	1.00E-96	1169423	bifunctional dihydrofolate reductase-thymidylate synthase	*Arabidopsis thaliana*
UI-D-GC0-aae-p-02-0-UI	1.00E-91	4416330	S-adenosyl-homocysteine hydrolase like protein	*Alexandrium fundyense*
UI-D-GC0-aae-h-11-0-UI	2.00E-91	21913167	oxygen evolving enhancer 1 precursor	*Heterocapsa triquetra*
UI-D-GC1-abh-d-23-0-UI	4.00E-91	32307578	glutamate 1-semialdehyde 2,1-aminomutase	*Bigelowiella natans*
UI-D-GC1-abe-e-15-0-UI	1.00E-89	27450753	proliferating cell nuclear antigen	*Pyrocystis lunula*
UI-D-GC1-abb-n-18-0-UI	3.00E-88	28277876	Similar to DEAD box polypeptide 48	*Danio rerio*
UI-D-GC1-aav-f-09-0-UI	3.00E-87	15240075	succinate dehydrogenase flavoprotein subunit, mitochondrial	*Arabidopsis thaliana*
UI-D-GC1-abc-o-16-0-UI	8.00E-85	13560096	ALA dehydratase	*Gonyaulax polyedra*
UI-D-GC1-aao-o-20-0-UI	1.00E-83	7025460	ADP ribosylation factor 1	*Toxoplasma gondii*
UI-D-GC0-aae-b-23-0-UI	5.00E-83	1076185	luciferin-binding protein	*Gonyaulax polyedra*
UI-D-GC1-aay-i-10-0-UI	9.00E-83	18416493	DEAD/DEAH box helicase, putative	*Arabidopsis thaliana*
UI-D-GC1-aau-b-16-0-UI	1.00E-82	4503507	eukaryotic translation initiation factor 2, subunit 3 gamma	*Homo sapiens*
UI-D-GC1-aad-d-15-0-UI	5.00E-81	1346753	Serine/threonine protein phosphatase PP1 isozyme 2	*Acetabularia cliftonii*
UI-D-GC1-aaz-h-12-0-UI	1.00E-77	23507885	26S proteasome regulatory subunit 4, putative	*Plasmodium falciparum*
UI-D-GC1-abc-m-19-0-UI	1.00E-77	32307576	geranyl-geranyl reductase	*Bigelowiella natans*
UI-D-GC1-abj-e-13-0-UI	2.00E-76	4033509	Calmodulin	*Tetrahymena pyriformis*
UI-D-GC1-abd-m-07-0-UI	3.00E-75	6831665	40S ribosomal protein S5	*Cicer arietinum*

As previously mentioned, our bioinformatic analyses identified 6,723 clusters of unique genes. However, this is likely to be a conservative estimate of the number of unique transcripts that were sequenced. A combination of short 3'-UTRs and highly conserved coding regions caused many related transcripts to be assembled together, even though their 3'-UTRs contained sequence differences. For example, two large clusters comprise ESTs that correspond to the plastid *atp*H gene that encodes the ATP synthase C chain. This gene is normally plastid encoded in other photosynthetic eukaryotes. These two clusters form closely related, but clearly distinct sets of transcripts. An additional *atp*H-encoding transcript was identified by a single EST. Together, the three clusters contain 43 ESTs, 16 of which are unique. The N-terminal extensions, which encode the tripartite plastid-targeting signals, share an average 74.3% nucleotide and 68.6% amino acid identity, respectively. Similar to many other species, the dinoflagellate transit peptides appear to be under selection to maintain hydrophobicity rather than a conserved amino acid sequence. This may explain why the nucleotide conservation is greater than that of the encoded amino acids. Five hydrophobic amino acids (phenylalanine, leucine, isoleucine, methionine, and valine) are, for example, encoded by codons with a T in the second position. This combined with the high GC-content at third positions results in higher conservation at second and third positions than at first positions. In addition, the high proportion of alanine (28.6%), leucine (10.2%), and valine (11.8%) rather than phenylalanine (2.4%), isoleucine (3.6%), methionine (4.3%, excluding starting methionine), and tyrosine (0.3%) in the N-terminal extensions may reflect the underlying GC-richness, because alanine, leucine, and valine are encoded by GC-rich codons. It is unclear if these amino acids are evolutionarily selected for specifically, or if they are selected for the combination of their hydrophobic character and the GC-content of their codons. In contrast, the conserved core of the protein shared an average 88.4% nucleotide and 98% amino acid identity, respectively, which corresponds to the more typical pattern of third position variation resulting from selection. The 3'-UTRs of the *atp*H genes show substantial variation and were difficult to align. There are several groups of more closely related 3'-UTRs that may be the result of recently duplicated genes. In all, there are five alignable groups of UTRs (and one singleton) that may have originated from more closely related genes.

### Histone and histone-like proteins in dinoflagellates

A significant finding of this study is the identification of two rare (2/11,171) ESTs that encode a partial histone H2A.X. The longest cDNA isolated from the library using PCR was predicted to encoded a protein of 169 amino acids that shares high sequence identity to eukaryotic histone H2A.X (Figure 3A). This clone putatively lacked only the start codon at the N-terminus. The divergent N-terminus of *A. tamarense *H2A.X is somewhat longer than in other homologs but the remainder of the sequence is conserved (in particular the α-helices of the histone fold). Several functional residues from the known crystal structure are also present in *A. tamarense *H2A.X including the lysine at the trypsin cleavage site, the arginines in the loops that interact with the DNA α-helix, and the lysine ubiquitination site [24]. The sites of interaction with histone H2B are also present.

**Figure 4:**
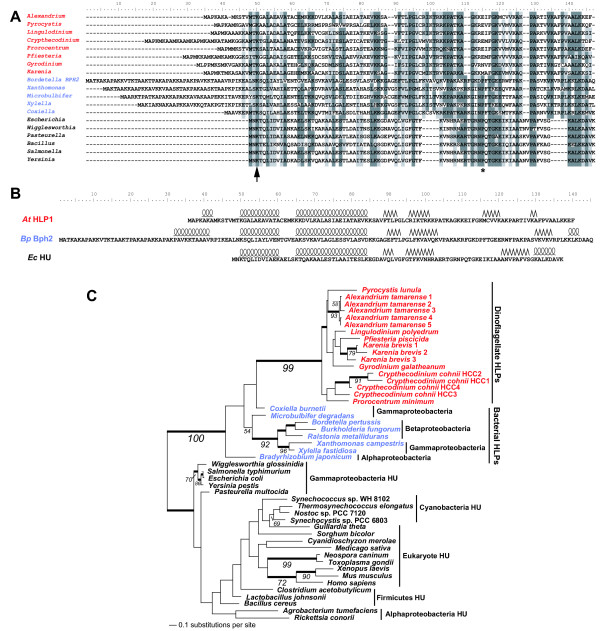
**Analysis of dinoflagellate HLPs. **A) HLPs from dinoflagellates (red taxa names) and bacteria (blue) and HU proteins from bacteria (black). B) The predicted secondary structure of HLPs from *A. tamarense *and *B. pertussis *aligned with the known secondary structure of *E. coli *HU. Curled lines indicate α-helices and jagged lines indicate β-strands. The arrow indicates the position of a conserved lysine. The asterisk indicates the proline that intercalates into the DNA in HU proteins. C) An ML tree of HU and HLP proteins from bacteria and eukaryotes. The numbers above and below the branches result from ML and NJ bootstrap analyses, respectively. The thick branches indicate > 0.95 posterior probability from Bayesian inference. Only bootstrap values ≥ 50% are shown. Branch lengths are proportional to the number of substitutions per site (see scale bar).

H2A.X proteins are closely related to the canonical H2A except for the C-terminus which contains the distinctive SQ(E/D)Φ motif (where Φ is a hydrophobic residue). H2A.X plays an important role in the recognition and repair of double-strand DNA breaks by non-homologous end-joining. At the site of double-strand breaks, the serine of the SQ(E/D)Φ motif is rapidly phosphorylated [25]. The phosphorylation signal spreads a large distance down the chromosome around the breaks, signalling the recruitment of the DNA repair proteins Rad50, Rad51, and BRCA1 [26,27]. We also identified histone H2A and H2A.X from the haptophyte *Emilania huxleyi *through high-throughput EST sequencing of this alga (J. D. H. and D. B. unpublished data). Phylogenetic analysis places *A. tamarense *H2A.X in its predicted position (with moderate bootstrap support) as sister to the *E. huxleyi *homolog within a group of chromalveolates that includes haptophytes, stramenopiles, and apicomplexans (Figure 3B). H2A.X from *A. tamarense*, *E. huxleyi*, and *Toxoplasma gondii *do not, however, form a monophyletic group suggesting multiple origins within chromalveolates. This is not surprising because H2A.X appears to have arisen independently many times during eukaryotic evolution [28,29].

We tested the strength of these results using the Approximately Unbiased (AU-) statistical test. A 16-taxon ML backbone tree was generated without *A. tamarense *H2A.X and then we made a set of 17-taxon trees by placing this sequence on every possible branch (29 in total). This analysis provides good support for the position shown in Figure 3B (P = 0.827), however, many alternative positions were included in the 5% confidence set of trees (i.e., as sister to *Thalassiosira pseudonana*, *Phaeodactylum tricornutum*, *Homo sapiens*, or *Drosophila melanogaster*, and at the base of or sister to either of the land plants). The lack of robust phylogenetic signal for the divergence point of *A. tamarense *H2A.X likely reflects the short length and high conservation of these histones.

Dinoflagellate chromosomes do not contain nucleosomes, instead the DNA is associated with HLPs [10,30,31]. The similarity between dinoflagellate HLPs and bacterial HU and HLPs has only recently been noted and these proteins have not yet been subjected to phylogenetic analysis with a broad taxon sampling [32]. In our *A. tamarense *EST data, HLPs were the most highly represented transcripts (45/11,171 ESTs) and encoded 5 closely related proteins. Alignment of the HLPs from *A. tamarense *and other dinoflagellates with HLPs and HU proteins from bacteria and eukaryotes showed moderate sequence similarity (a representative alignment is shown in Figure 4A). This alignment was constructed using information from secondary structure predictions (discussed below).

One group of proteins (referred to here as bacterial HLPs) is more closely related to dinoflagellate HLPs and includes Bph2 from *Bordetella pertussis*. Bph2 has a role in virulence gene expression and shares limited (likely convergent) sequence similarity with histone H1 [33]. The dinoflagellate and bacterial HLPs also contain an N-terminal extension in comparison to HU proteins. This extension is rich in alanine, lysine, and proline, which is reminiscent of the C-terminus of histone H1. The dinoflagellate HLP N-termini are however, also enriched in methionines. Compared to the bacterial HLPs, this N-terminal region is generally shorter in the dinoflagellates, although there is variability among species in both groups (Figure 4A). In contrast to the primary sequence, secondary structure predictions for these three classes of proteins are remarkably similar. The crystal structure of *E. coli *HU has been determined (PBD ID: 1MUL) and the known secondary structure was compared to the predicted secondary structures of *B. pertussis *Bph2 and an *A. tamarense *HLP (Figure 4B). Both types of HLPs are predicted to have two α-helices that are identical in size and spacing to the N-terminal helices in *E. coli *HU, followed by two β-strands that are similar in size and position. We conclude from this analysis that dinoflagellate HLPs show structural similarity to HU proteins from bacteria, however, it is unclear if these proteins are functional homologs. It is also apparent that dinoflagellate HLPs are distantly related to bacterial HU proteins. The dinoflagellates have one putatively homologous functional residue corresponding to Lys3 (arrow in Figure 4A) of HU proteins, which interacts with the DNA and is involved in wrapping the DNA around the protein [34]. A proline residue (asterisk in Figure 4A), which intercalates into the DNA during HU binding, appears to be conserved among HU proteins and bacterial HLPs, but is not present in the dinoflagellate HLPs [35]. However, there are several prolines conserved among dinoflagellates in the C-terminal end of the protein. The C-terminal arms of HU are critical for the interactions that bend the DNA. Given the low level of sequence similarity and the absence of a homologous proline in this region, it is unclear if the dinoflagellate HLPs are able to interact with DNA in the same manner as HU proteins.

In our phylogenetic analyses, the proteobacterial HLPs form a well-supported monophyletic group with the dinoflagellates (Figure 4C) suggesting an origin of the dinoflagellate gene through lateral transfer (followed by several rounds of gene duplication). It is also noteworthy that dinoflagellates are the only eukaryotes to possess a proteobacterial form II rubisco [36]. The position of the dinoflagellate HLPs is distinct from that of other eukaryotic HU proteins. These latter proteins group with the canonical HU proteins from bacteria and have likely originated through intracellular transfer from the mitochondrial or plastid endosymbiont. Statistical support for the monophyly of the dinoflagellate and proteobacterial HLPs was tested using the AU-test. In these analyses (details not shown), a sister group relationship between the HLPs was the most highly favored topology (P = 0.659) and all other positions for the dinoflagellates (except branching inside the bacterial HLP clade) had significantly lower probabilities (P < 0.05).

Dinoflagellates no longer use the nucleosome as the major DNA packaging protein complex. Chromosomal DNA strands in these taxa are smooth, in contrast to the "beads on a string" conformation in other eukaryotes [12]. The chromosome structure is also unique in that they are uniform in size and morphology, remain condensed throughout the cell cycle, and are birefringent, indicating a liquid crystal state [5,14,37]. Transcription is thought to take place in DNA loops that protrude from the condensed chromosome [38]. It appears that dinoflagellates have acquired DNA binding proteins from a proteobacterium possibly to facilitate the compaction of their immense genomes. HU and related proteins from bacteria induce sharp bends in DNA strands and some models suggest that HLPs are responsible for creating DNA bends at the periphery of the chromosomes [39,40]. Immunolocalization shows dinoflagellate HLP to be associated with the periphery of chromosomes [41].

However, the HLP concentration is very low relative to DNA content. Dinoflagellate chromosomes have a 1:10 protein:DNA ratio (in contrast to the 1:1 ratio in other eukaryotes). The HLP concentration may therefore be too low to function in DNA compaction, rather they may act as transcriptional regulators [41,42].

In summary, our discovery of H2A.X in *A. tamarense *shows that, whereas dinoflagellates appear to no longer use nucleosomes for DNA packaging, at least one histone has been retained and is weakly expressed. Interestingly, in a recent paper, histone H3 appears in a table of redox-regulated genes in the dinoflagellate *Pyrocystis lunula *[11]. Until now, only these two histones have been identified in dinoflagellates and it is unclear if all dinoflagellates possess either of these two genes, or others that have not yet been found. If other histones are present (which is likely), they may however also be expressed at a low level (as is the case for *A. tamarense *H2A.X). This would render difficult their identification using the EST-based approach unless comprehensive sequencing of normalized and subtracted cDNA libraries is used. In metazoans, replication-dependant canonical histone (H2A, H2B, H3 and H4) mRNAs are not polyadenylated, raising the possibility that they have been excluded from this poly-A primed cDNA library [43]. However, these histone mRNAs are polyadenylated in plants, apicomplexans, and ciliates, suggesting that if they are present, they may be in dinoflagellates as well [44-46]. Given the important role that H2A.X plays in DNA repair, we speculate that this gene may have been maintained specifically to perform this function. Consistent with this idea, the core region of *A. tamarense *H2A.X is highly conserved, indicating that it may still be able to interact with DNA in a manner similar to H2A in other species.

## Conclusion

This collection of ESTs is the most extensive genomic resource for a toxic dinoflagellate species to date and provides a useful glimpse into its nuclear genome. These data will be instrumental to future research to understand the unique and complex cell biology of these organisms and for understanding the method of toxin production in these species. We have likely not yet exhausted the gene discovery potential using the EST approach (i.e., note the high discovery rate of our normalized library). In the future, we will use serial subtraction of cDNA libraries to improve/maintain the novelty rate of our cDNA library and create cDNA libraries from *A. tamarense *under various growth conditions and life history stages to get generate a more complete catalog of the gene content of this important organism.

## Methods

### Library construction

Total RNA from a culture of the toxic dinoflagellate *Alexandrium tamarense *(CCMP 1598) was extracted using Trizol (GibcoBRL) and mRNA purified using the Oligotex mRNA Midi Kit (Qiagen). This culture strain was produced by isolating a single cyst, a diploid resting stage that produces haploid vegetative cells by meiosis. However, it is unknown if a single or multiple vegetative cells were isolated after antibiotic treatment to make the culture axenic. If a single vegetative cell was isolated, the culture would be clonal. The culture was grown at 20°C on a 13:11 hour light:dark cycle (80 μEinsteins of light) in L1 media. Start and normalized directionally cloned (3' NotI-5'EcoR1) cDNA libraries were constructed as previously described [47]. ESTs were sequenced from the 3' end to maximize clustering accuracy using the 3' untranslated region (UTR). All ESTs were processed as previously described [48]. Identification of a total of a non-redundant "unigene" set of 6,723 unique clusters from 11,171 sequences was accomplished using using UIcluster v3.0.5 [49].

### Phylogenetic analyses

Data was gathered from GenBank (including the recently released *Karenia brevis *EST data, Frances Van Dolah, unpublished data) using blast searches. Maximum likelihood (ML) analyses were done with PHYLIP using the JTT model of protein evolution with gamma corrected rates (JTT + Γ) with 5 random additions [50]. ML bootstrap analyses (100 replications) were done as described with either 5 (histone H2A) or 1 (HLPs) rounds of random taxon addition. Bayesian analyses were done using MrBayes V3.0b4 [51]. Four chains (1 cold, 3 heated) were run for 1 million generations, sampled every 1000 generations, using the JTT + Γ model. The first 500 trees were discarded as burn-in. Neighbor joining (NJ) bootstrap (500 replicates) analyses were done with PHYLIP using the JTT + Γ model. Minimum evolution (ME) analyses done with PHYLIP using the JTT + Γ model with global rearrangements and 10 rounds of random taxon addition (1 round was used in the bootstrap analysis).

The Approximately Unbiased test was done using CONSEL [52]. ML trees without the groups of interest were generated as described above. A pool of trees was then generated by adding the group of interest (*A. tamarense *H2A.X or dinoflagellate HLPs) to every possible branch in the ML tree. For the HLP analyses, a reduced taxon set was used that included *Bordetella*, *Ralstonia*, *Xylella*, *Pasteurella*, *Nostoc*, *Synechocystis*, *Agrobacterium*, *Rikettsia*, *Escherichia*, *Guillardia*, *Cyanidioschyzon*, *Sorghum*, *Toxoplasma*, *Xenopus*, and *Homo*. *A. tamarense *1 and *C. cohnii *HCC2 were added as a monophyletic group to every branch in this reduced ML tree. Secondary structure prediction was done using Jpred [53, 54]. The consensus secondary structures were used in the comparison to the know structure of E. coli HU (PDB ID: 1MUL).

## Authors' contributions

JDH constructed the cDNA libraries and did the sequence and phylogenetic analyses, the Blast and GO analyses on the EST dataset, the histone and HLP analyses, and drafted the manuscript. TES did many of the other global sequence analyses of the EST dataset. HSY contributed intellectually to the manuscript. Library construction and high-throughput EST sequencing was done in the laboratory of MBS and was supervised by MFB. The bioinformatics group led by TLC did the EST sequence processing and clustering. DB conceived of and supervised this study and contributed to the manuscript. All authors read and approved the final manuscript.

## References

[B1] Hackett JD, Anderson DM, Erdner DL, Bhattacharya D (2004). Dinoflagellates: A remarkable evolutionary experiment.. American Journal of Botany.

[B2] Graham LE, Wilcox LW (2000). Algae.

[B3] Hallegraeff GM (1993). A review of harmful algal blooms and their apparent global increase.. Phycologia.

[B4] Trench RK, Taylor FJR (1987). Dinoflagellates in non-parasitic symbioses. The Biology of Dinoflagellates.

[B5] Dodge JD, Godward MBE (1966). The Dinophyceae. The chromosomes of the algae.

[B6] Oakley B, Dodge JD (1974). Kinetochores associated with the nuclear envelope in the mitosis of a dinoflagellate,. Journal of Cell Biology.

[B7] Rizzo PJ (1991). The enigma of the dinoflagellate chromosome. Journal of Protozoology.

[B8] Rizzo PJ (1981). Comparative aspects of basic chromatin proteins in dinoflagellates. Biosystems.

[B9] Wong JTY, New DC, Wong JCW, Hung VKL (2003). Histone-Like Proteins of the Dinoflagellate Crypthecodinium cohnii Have Homologies to Bacterial DNA-Binding Proteins. Eukaryotic Cell.

[B10] Rizzo PJ (2003). Those amazing dinoflagellate chromosomes. Cell Res.

[B11] Okamato OK, Hastings JW (2003). Genome-wide analysis of redox-regulated genes in a dinoflagellate. Gene.

[B12] Spector DL, Spector DL (1984). Dinoflagellate Nuclei. Dinoflagellates.

[B13] Livolant F, Bouligand Y (1978). New observations on the twisted arrangement of dinoflagellate chromosomes.. Chromosoma.

[B14] Gautier A, Michel-Salamin L, Tosi-Couture E, McDowall AW, Dubochet J (1986). Electron microscopy of the chromosomes of dinoflagellates in situ: confirmation of Bouligand's liquid crystal hypothesis.. Journal of Ultrastructure and Molecular Structure Research.

[B15] Rae PMM (1976). Hydroxymethyluracil in eukaryote DNA: A natural feature of the Pyrrophyta (Dinoflagellates).. Science.

[B16] Zhang Z, Green BR, Cavalier-Smith T (1999). Single gene circles in dinoflagellate chloroplast genomes. Nature.

[B17] Barbrook AC, Howe CJ (2000). Minicircular plastid DNA in the dinoflagellate Amphidinium operculatum. Mol Gen Genet.

[B18] Hackett JD, Yoon HS, Soares MB, Bonaldo MF, Casavant TL, Scheetz TE, Nosenko T, Bhattacharya D (2004). Migration of the plastid genome to the nucleus in a peridinin dinoflagellate. Curr Biol.

[B19] Bachvaroff TR, Concepcion GT, Rogers CR, Herman EM, Delwiche CF (2004). Dinoflagellate expressed sequence tag data indicate massive transfer of chloroplast genes to the nuclear genome. Protist.

[B20] Nassoury N, Cappadocia M, Morse D (2003). Plastid ultrastructure defines the protein import pathway in dinoflagellates.. Journal of Cell Science.

[B21] Schnepf E, Elbrachter M (1999). Dinophyte chloroplasts and phylogeny - A review. Grana.

[B22] Mazumder B, Seshadri V, Fox PL (2003). Translational control by the 3'-UTR: the ends specify the means. Trends in Biochemical Sciences.

[B23] Bhattacharya D, Yoon HS, Hackett JD (2004). Photosynthetic eukaryotes unite: endosymbiosis connects the dots. Bioessays.

[B24] Luger K, Mader AW, Richmond RK, Sargent DF, Richmond TJ (1997). Crystal structure of the nucleosome core particle at 2.8 A resolution. Nature.

[B25] Rogakou EP, Pilch DR, Orr AH, Ivanova VS, Bonner WM (1998). DNA double-stranded breaks induce histone H2AX phosphorylation on serine 139. J Biol Chem.

[B26] Rogakou EP, Boon C, Redon C, Bonner WM (1999). Megabase chromatin domains involved in DNA double-strand breaks in vivo. J Cell Biol.

[B27] Paull TT, Rogakou EP, Yamazaki V, Kirchgessner CU, Gellert M, Bonner WM (2000). A critical role for histone H2AX in recruitment of repair factors to nuclear foci after DNA damage. Curr Biol.

[B28] Thatcher TH, Gorovsky MA (1994). Phylogenetic analysis of the core histones H2A, H2B, H3, and H4. Nucleic Acids Res.

[B29] Malik HS, Henikoff S (2003). Phylogenomics of the nucleosome. Nat Struct Biol.

[B30] Allen JR, Roberts M, Loeblich AR, Klotz LC (1975). Characterization of the DNA from the dinoflagellate Crypthecodinium cohnii and implications for nuclear organization. Cell.

[B31] Wong JT, New DC, Wong JC, Hung VK (2003). Histone-like proteins of the dinoflagellate Crypthecodinium cohnii have homologies to bacterial DNA-binding proteins. Eukaryot Cell.

[B32] Goyard S (1996). Identification and characterization of BpH2, a novel histone H1 homolog in Bordetella pertussis. J Bacteriol.

[B33] Grove A, Saavedra TC (2002). The role of surface-exposed lysines in wrapping DNA about the bacterial histone-like protein HU.. Biochemistry.

[B34] Swinger KK, Lemberg KM, Zhang Y, Rice PA (2003). Flexible DNA bending in HU–DNA cocrystal structures. EMBO Journal.

[B35] Morse D, Salois P, Markovic P, Hastings JW (1995). A nuclear-encoded form II RuBisCO in dinoflagellates. Science.

[B36] Loeblich AR (1976). Dinoflagellate evolution: speculation and evidence. J Protozool.

[B37] Soyer-Gobillard MO, Geraud ML, Coulaud D, Barray M, Theveny B, Revet B, Delain E (1990). Location of B- and Z-DNA in the chromosomes of a primitive eukaryote dinoflagellate. J Cell Biol.

[B38] Sandman K, Pereira SL, Reeve JN (1998). Diversity of prokaryotic chromosomal proteins and the origin of the nucleosome. Cell Mol Life Sci.

[B39] Bouligand Y, Norris V (2001). Chromosome separation and segregation in dinoflagellates and bacteria may depend on liquid crystalline states. Biochimie.

[B40] Sala-Rovira M, Geraud ML, Caput D, Jacques F, Soyer-Gobillard MO, Vernet G, Herzog M (1991). Molecular cloning and immunolocalization of two variants of the major basic nuclear protein (HCc) from the histone-less eukaryote Crypthecodinium cohnii (Pyrrhophyta). Chromosoma.

[B41] Chudnovsky Y, Li JF, Rizzo PJ, Hastings JW, Fagan TF (2002). Cloning, expression, and characterization of a histone-like protein from the marine dinoflagellate Lingulodinium polyedrum (Dinophyceae).. Journal of Phycology.

[B42] Dominski Z, Marzluff WF (1999). Formation of the 3' end of histone mRNA. Gene.

[B43] Chaboute ME, Chaubet N, Gigot C, Philipps G (1993). Histones and histone genes in higher plants: structure and genomic organization. Biochimie.

[B44] Liu X, Gorovsky MA (1996). Cloning and characterization of the major histone H2A genes completes the cloning and sequencing of known histone genes of Tetrahymena thermophila.. Nucleic Acids Research.

[B45] Rawat DS, Sharma I, Jalah R, Lomash S, Kothekar V, Pasha ST, Sharma YD (2004). Identification, expression, modeled structure and serological characterization of Plasmodium vivax histone 2B. Gene.

[B46] Bonaldo MF, Lennon G, Soares MB (1996). Normalization and subtraction: two approaches to facilitate gene discovery. Genome Res.

[B47] Scheetz TE, Laffin JJ, Berger B, Holte S, Baumes SA, Brown R, Chang S, Coco J, Conklin J, Crouch K, Donohue M, Doonan G, Estes C, Eyestone M, Fishler K, Gardiner J, Guo L, Johnson B, Keppel C, Kreger R, Lebeck M, Marcelino R, Miljkovich V, Perdue M, Qui L, Rehmann J, Reiter RS, Rhoads B, Schaefer K, Smith C, Sunjevaric I, Trout K, Wu N, Birkett CL, Bischof J, Gackle B, Gavin A, Grundstad AJ, Mokrzycki B, Moressi C, O'Leary B, Pedretti K, Roberts C, Robinson NL, Smith M, Tack D, Trivedi N, Kucaba T, Freeman T, Lin JJ, Bonaldo MF, Casavant TL, Sheffield VC, Soares MB (2004). High-throughput gene discovery in the rat. Genome Res.

[B48] Trivedi N, Bischof J, Davis S, Pedretti K, Scheetz TE, Braun TA, Roberts CA, Robinson NL, Sheffield VC, Soares MB, Casavant TL (2002). Parallel creation of non-redundant gene indices from partial mRNA transcripts. Future Generation Computer Systems.

[B49] Felsenstein J (2002). PHYLIP (Phylogeny Inference Package) version 3.6 (Department of Genetics, University of Washington, Seattle, WA)..

[B50] Huelsenbeck JP, Ronquist F (2001). MrBayes: Bayesian inference of phylogenetic trees. Bioinformatics.

[B51] Shimodaira H, Hasegawa M (2001). CONSEL: for assessing the confidence of phylogenetic tree selection.. Bioinformatics.

[B52] Cuff JA, Clamp ME, Siddiqui AS, Finlay M, Barton GJ (1998). Jpred: A Consensus Secondary Structure Prediction Server. Bioinformatics.

[B53] Jpred: A consensus secondary structure prediction server.

